# A concentric tube-based 4-DOF puncturing needle with a novel miniaturized actuation system for vitrectomy

**DOI:** 10.1186/s12938-019-0666-x

**Published:** 2019-04-18

**Authors:** Muhammad Umar Farooq, Binxiang Xu, Seong Young Ko

**Affiliations:** Department of Mechanical Engineering, Chonnam National University, Gwangju, 61186 South Korea

**Keywords:** Concentric tube robots (CTR), Vitrectomy, Ophthalmic surgery, Puncturing needle

## Abstract

**Background:**

Vitreoretinal surgeries require precise, dexterous, and steady instruments for operation in delicate parts of the eye. Robotics has presented solutions for many vitreoretinal surgical problems, but, in a few operations, the available tools are still not dexterous enough to carry out procedures with minimum trauma to patients. Vitrectomy is one of those procedures and requires some dexterous instruments to replace straight ones for better navigation to affected sides inside the eyeball.

**Method:**

In this paper, we propose a new vein puncturing solution with a 4-DOF motion to increase the workspace inside the eye. A two-member concentric tube-based 25G needle is proposed whose shape is optimized. To operate the concentric tube needle, a novel and miniaturized actuation system is proposed that uses hollow shaft motors for the first time. The presented prototype of actuation system has a stroke of 100 mm in a small size of 148 × 25 × 65 mm (*L* × *W* × *H*), suitable for approaching distant positions inside the eyeball.

**Results:**

Experimental results validate that the targeting accuracy of the needle is less than one millimeter and the needle tip can apply a force of 23.51 mN which is enough to perform puncturing. Furthermore, the proposed needle covers maximum workspace of around 128.5° inside the eyeball. For the actuation system, experiments show that it can produce repeatable motions with accuracy in submillimeter.

**Conclusion:**

The proposed needle system can navigate to the sites which are difficult to approach by currently available straight tools requiring reinsertions. Along with the miniaturized actuation system, this work is expected to improve the outcome of vitrectomy with safe and accurate navigation.

## Background

Eye, one of the most delicate parts of the human body, requires steady and accurate tools with enough dexterity to cause the least trauma to patients. Vitreoretinal surgery is a part of ophthalmic surgery that deals with the issues related to the retina. Due to inflammation in the retinal arteries and veins, clouding of vitreous fluid (gel-like substance present behind the lens of the eyes), and swelling of the retina, vision is severely obscured resulting in uveitis [1]. This kind of intraocular inflammation is a significant cause of vision loss despite the current developments in the treatment of uveitis [2]. Vitrectomy is performed to treat uveitis and intraocular inflammation. The surgical procedure involves puncturing of swollen retinal arteries and veins and removal of infectious fluid along with the vitreous fluid [1]. The available dexterity of the tools used for vitrectomy is limited due to their straight nature. Repeated insertions are required to reach different surgical targets which increase the incision size necessitating post-operative stitching. A fulcrum motion is generated when a tool is inserted in the eyeball causing stress on the sclera (white region of the eye). This stress is a cause of trauma and should be minimum, but it is highly dependent on the number of incisions which are commonly high with straight tools. Reaching behind the retina and accessing distant targets are also challenging with such straight tools that lack suitable maneuverability. In addition, the visual details of the operating tool inside the eyeball are limited under microscopic guidance during the surgical procedure. The eye of the patient is pushed out for a clearer view which increases trauma. Hence, a sufficiently dexterous tool may reduce the complexity of the procedure for the surgeons and decrease trauma for patients.

### Puncturing needle

Robotics has provided viable solutions to many clinical issues for almost three decades [3] and first robotic system for ophthalmic surgery was a 6-DOF stereotaxic micro-telemanipulator (SMOS) introduced in 1989 [4]. Concentric tube robots (CTR); a type of continuum robots, are composed of elastic and pre-curved tubes nested inside each other and moved by the relative rotation and translation of tubes [5]. CTR is keenly investigated these days for use in surgical procedures due to their dexterous nature and ease of miniaturization [6]. In ophthalmic surgeries, 23G CTR-based grippers have been introduced before for use in vitreoretinal surgery [7]. They can cover a workspace of around 110° inside the eyeball.

Most of the tools available to date for tasks like puncturing and vein cannulation are straight using 30G needles with a catheter [8]. The da Vinci system is somewhat feasible for post-operative suturing in extraocular surgeries, but its use in intraocular surgeries has some issues and researchers are still testing its efficacy in ophthalmic procedures [4]. A 3-DOF membrane peeling solution is introduced in [9] which can measure forces with a resolution in submillinewton at the distal end. It is non-backdrivable with a remote center-of-motion (RCM) mechanism. An intraocular snake of 19G (0.9 mm) is used for retinal vein cannulation in [10]. It uses the variable neutral-line mechanism and can have bending of around 100°. IRIS (Integrated Robotic Intraocular Snake) has a 2-DOF body and provides 2-DOF motion at the distal end for the surgical tools. The diameter of [10] is high and requires suturing after surgery with only 2-DOF motion, while, in [9], the tool lacks required dexterity for maneuvering to hard to reach targets because of its straight nature and has the same diameter with [10].

### Actuation system

CTR is almost a decade old in the field of robotics and clinically applicable actuation units are required for their acceptance in the surgical room [6]. Different actuation systems have been developed already according to surgical requirements. A piezoelectric actuation system for a two-member CTR is presented in [11] that lowers the number of artifacts in MR image during robot operation. A manual and autoclavable actuation system with modular and sterilizable design is introduced in [12]. In [13], a joystick-operated handheld actuation device for steerable needle was developed. It weighed around 250 g with 20 mm axial motion and unlimited rotation on the stylet. A telerobotic system for trans-nasal access [14] was also developed for CTR. Recently, a compact actuation system is developed that utilizes waffle gears [15] and can also be used as a handheld device, but has a backlash problem. Our group also developed an actuation system [16] for CTR that is more miniaturized than [15]. The key part of our previously developed version was the compact and cost-effective design using only two actuators to drive six members (which is the maximum number of CTR members reported to date [6]). The actuation system was sequential and supported an only translational motion for six members. The proposed needle system has two members that require both translation and rotation for operation inside the eye. Therefore, a new task-specific actuation system is required. The other actuation systems present in the literature are bulky, complex, and use expensive materials. A more compact and cost-effective solution may be redeemed that does not compromise the performance and degrees of freedom of the robot.

### Contributions

A 4-DOF needle based on the CTR approach for puncturing and vein cannulation is presented in this paper. It consists of two pre-curved segments; a cannula and stylet, and the shape of whole CTR is optimized to cover more workspace inside the eye and disallow repeated insertions. A novel miniaturized actuation system for 4-DOF CTR is also presented here which uses hollow shaft stepper motors for the first time. The developed actuation system is repeatable and provides two degrees of freedom (translation and rotation) for each member. The contribution of this paper is: (1) a new 4-DOF concentric tube needle to carry out puncturing and vein cannulation in vitrectomy, (2) optimized design to cover more workspace inside the eyeball, and (3) a novel and miniaturized actuation system for CTR using hollow shaft motors.

## Methods

### Puncturing needle

#### Design

**Fig. 1:**
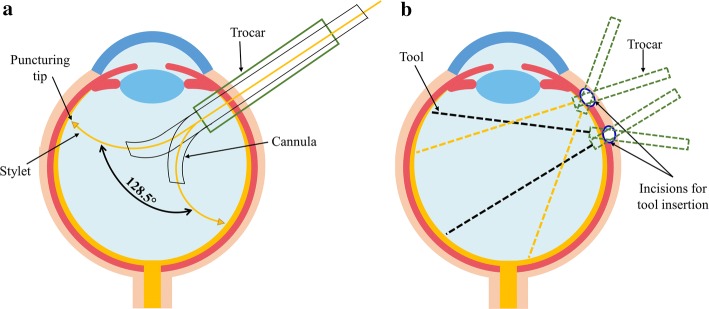
Schematic diagram of the motion of the presented pre-bent tool vs the conventional straight one inside the eyeball; **a** advantage of the bendable tool; **b** limitations of a straight tool

The currently available straight tools have an issue of repeated reinsertions if a new target has to be reached inside the eyeball. In some cases, surgeons push out the eye of the patient to reach distant targets causing pain. Such actions will cause trauma to patients and necessitate stitching for scleral closure. Thus, a tool that can turn inside the eyeball without moving its initial trocar position would be more desirable. Figure 1 explains the advantage of bendable tools over the straight ones. The straight tool must be reinserted, and some targets are still hard to reach without colliding sensitive parts of the eye, while curved tools can turn inside the eyeball without causing any stress on sclera with an ability of collision avoidance and reaching more distant targets.

**Fig. 2:**
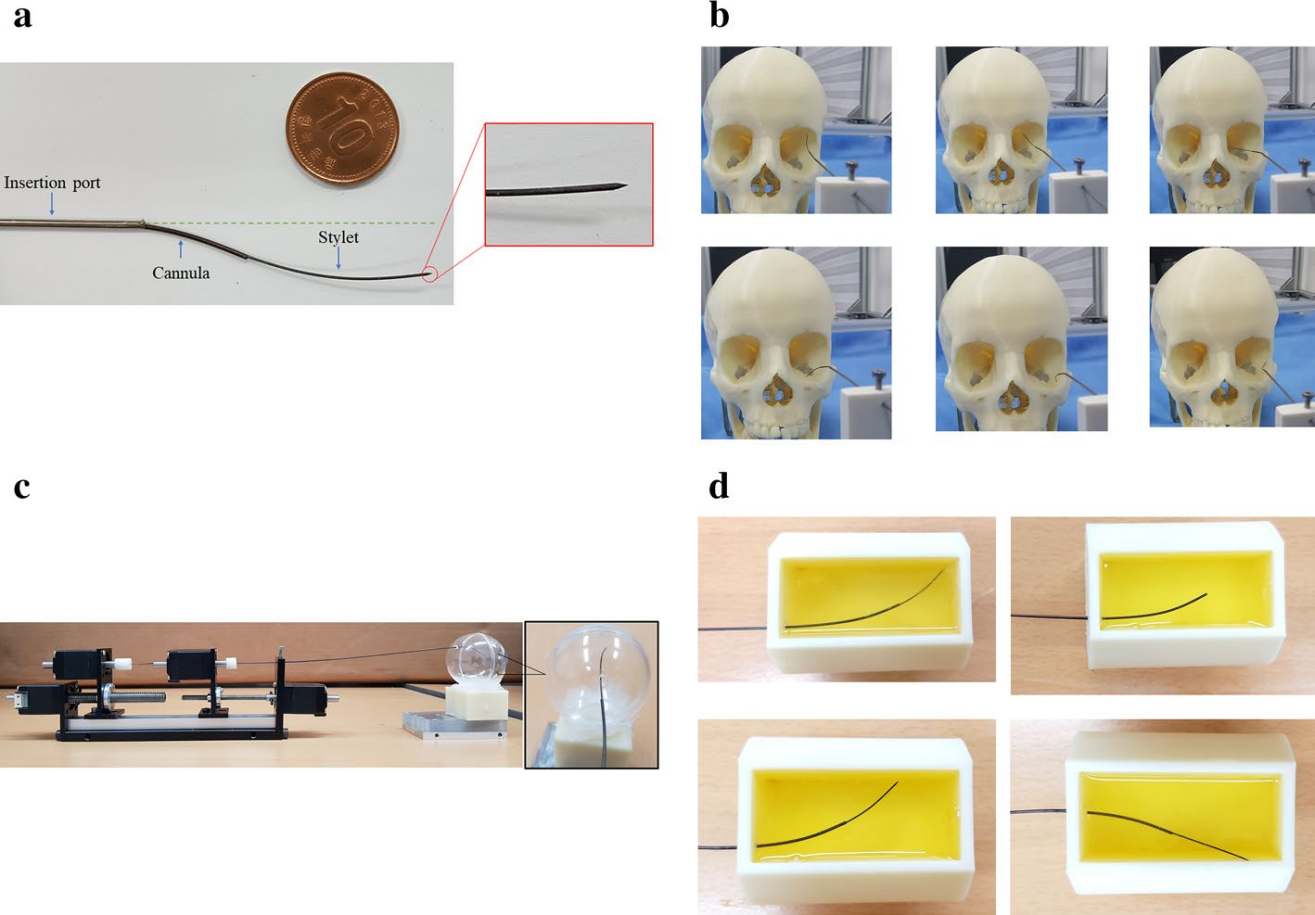
Feasibility of proposed needle system in different conditions; **a** developed prototype with a 10 KRW coin for size reference (10KRW coin has a diameter of 22.86 mm). The inset view shows the puncturing tip on the 25G stylet, **b** movement of needle inside the eyeball of skull phantom to reach different targets from the same insertion point, **c** the proposed needle system inside a phantom eyeball, and **d** movement of the needle inside a block that is filled with albumen (white part of the egg)

To achieve that goal, we propose a 4-DOF needle system shown in Fig. 2a. The whole structure of the proposed puncturing needle should make an S-curved shape inside the eye for easy maneuvering, bigger workspace, and easy access to hard to reach surgical targets. As the needle was developed using the CTR approach, the whole needle is divided into two segments for stability; cannula and stylet. The 4-DOF design is developed to provide a better reachable workspace. The cannula and stylet can be rotated and translated relative to each other to cover more workspace and generate a new pose to reach distant targets inside the eye. In addition, the 4-DOF and two-member design will provide the possibility to carry out drug delivery tasks. The stylet can be used to puncture the veins, while cannula can be used as a vessel for on-site drug delivery. The whole CTR was nested inside a nitinol insertion port of outer and inner diameters of 1.3 mm and 1.1 mm, respectively. Figure 2b shows the advantage of CTR-based needle inside the eyeball of skull phantom, while Fig. 2c shows the working of the needle inside the eyeball phantom. The developed prototype was also tested in the albumen (white part of the egg) which mimics the properties of vitreous fluid present in the human eye. The needle system was aimed at different targets to show the applicability of the device.

#### Optimization

To reach distal targets situated behind the retina, the curved length of the whole needle structure was fixed to 45 mm (to reach the end point of the eyeball). Both cannula and stylet are nitinol tube and wire, respectively, and their shape was optimized in MATLAB^®^. A surgical target was chosen that is situated 45 mm away from the insertion point. The shape of the segments was solved using the approach presented in [17]:

1 $$\rho \left( s \right) = \left\{ {\begin{array}{ll} a, &\quad 0 < s \le s_{1} \\ b,&\quad s_{1} < s \le s_{2} \\ \end{array} } \right.$$

2 $$\theta \left( s \right) = \theta_{0} + \mathop \int \limits_{0}^{s} p\left( \tau \right){\text{d}}\tau$$

3 $$x\left( s \right) = x_{0} + \mathop \int \limits_{0}^{s} \cos \left( {\theta \left( \tau \right)} \right){\text{d}}\tau$$

4 $$y\left( s \right) = y_{0} + \mathop \int \limits_{0}^{s} \sin \left( {\theta \left( \tau \right)} \right){\text{d}}\tau .$$

**Fig. 3:**
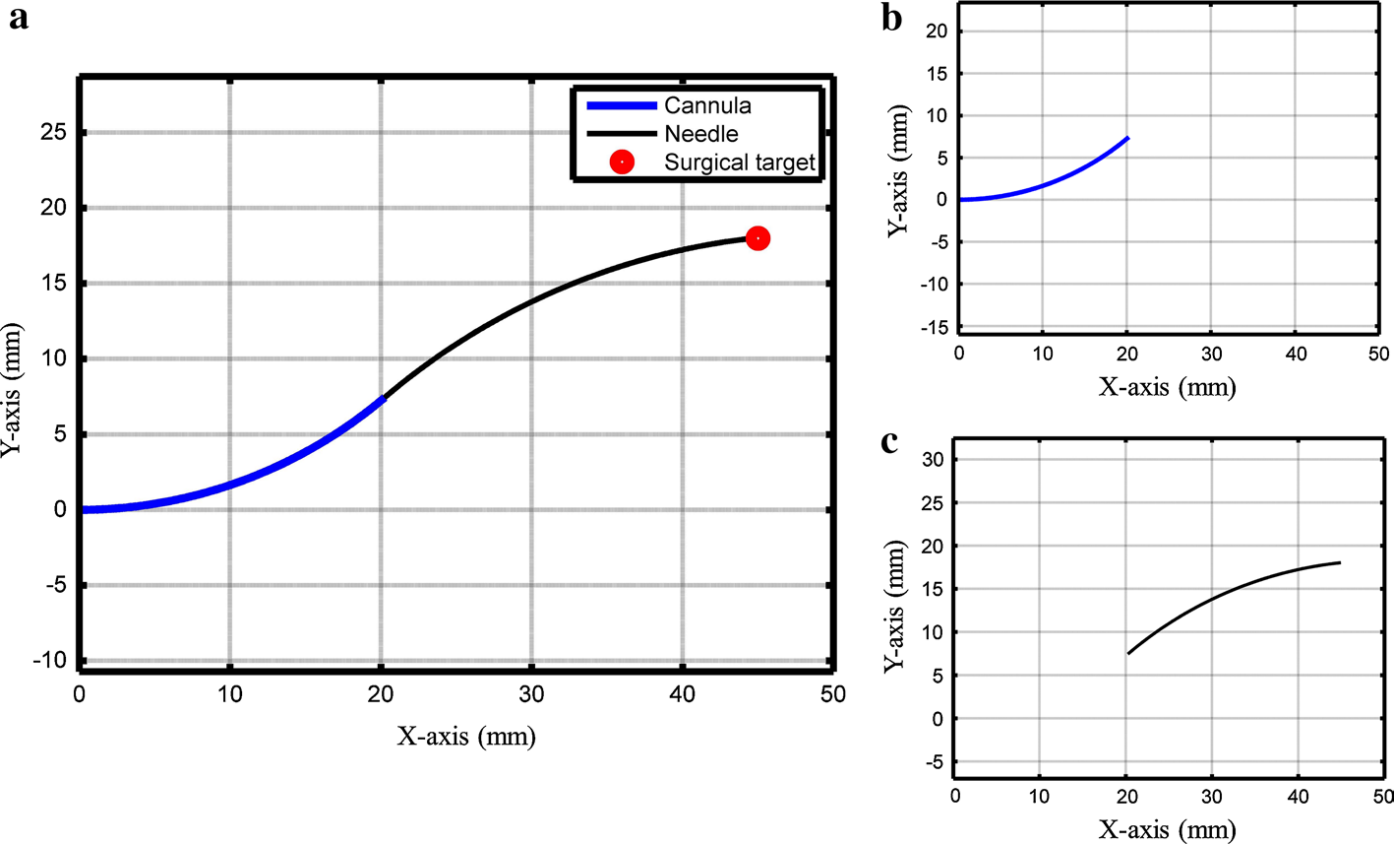
Optimized shape of the needle; **a** total shape, **b** cannula, and **c** stylet

Here, *θ*_0_, *x*_0_, and *y*_0_ are initial projection angle and 2D position of the insertion point, respectively. The curvature and the arc length of cannula and stylet are *a*, *b s*_1_, and *s*_2_, respectively. Equation 1 is the curvature function and Eq. 2 is the angle function, while Eqs. 3 and 4 is the position of the whole needle in 2D space. The geometrical parameters for insertion point [0,0], endpoint of cannula [20,7.5], and surgical target [45,18] were provided in the MATLAB program, and the whole system was solved for the values of curvature and arc length (using ‘fminsearch’ solver in optimization toolbox. Detailed information on the working of shape optimization algorithm is already presented in [17]). These solved parameters along with the length and diameter of needle segments are provided in Table 1, while the optimized shape is shown in Fig. 3.

**Table 1:** Parameters of needle segments

Member	OD (mm)	ID (mm)	*K* (mm^−1^)	*s* (mm)	*L* (mm)
Cannula	0.8128	0.7112	0.0320	22.1370	200
Stylet	0.46	–	− 0.0220	49.5387	300

#### Development

As the needle segments are of nitinol, shape setting process was applied to achieve the pre-curved structure. Using the optimized curvature and arc length values, an aluminum jig was developed to constrain the segments. An air furnace was used to heat the nitinol segments at 550 °C at a heating rate of 10 °C/min. The segments were kept at the final temperature for 15 min and cold-water quenching was performed afterward. To make the puncturing tip on stylet, nitinol wire was laser cut in a triangular shape with vertices angle of 45°.

### Actuation system

#### Design and development

**Fig. 4:**
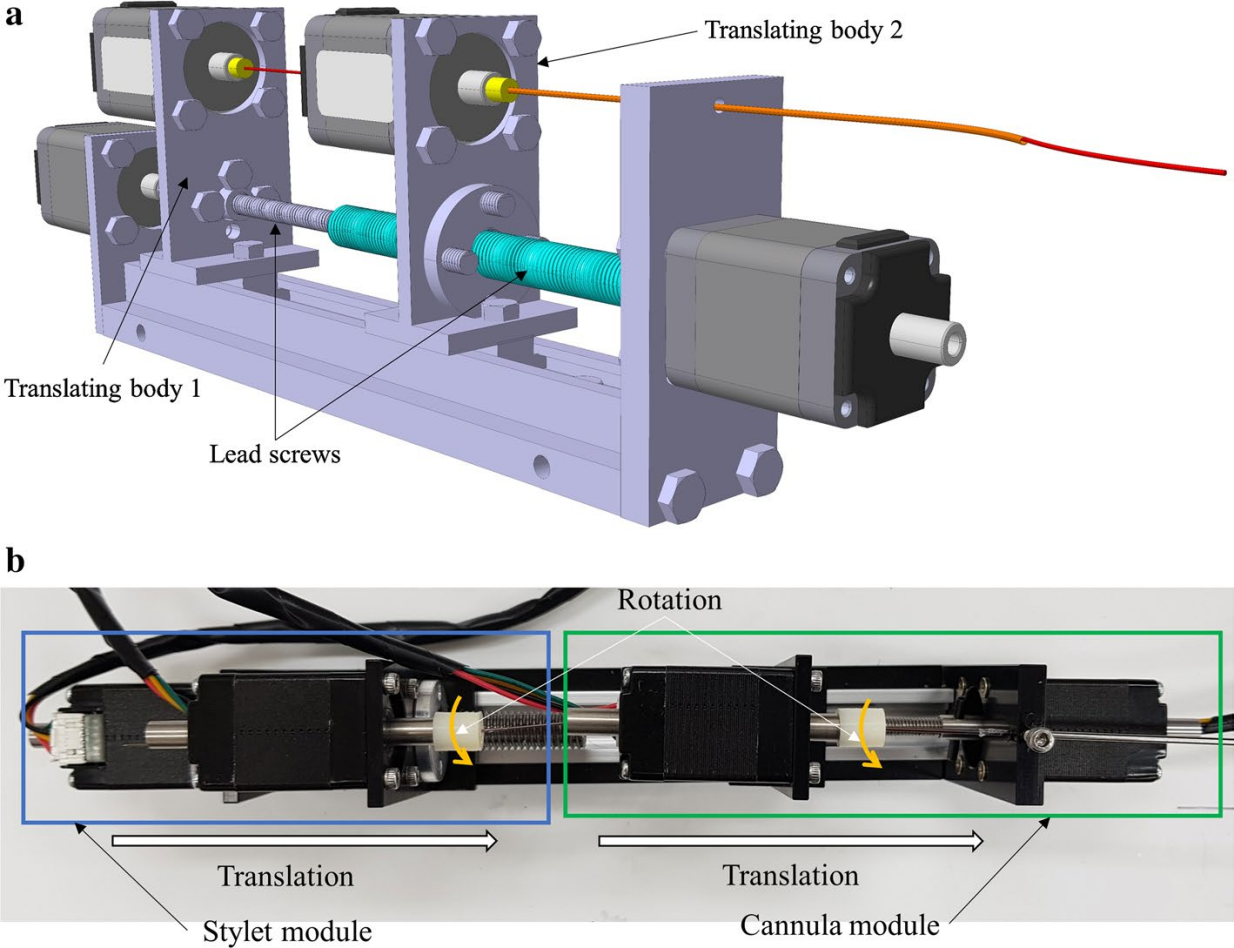
Developed prototype of the hollow shaft motor-based actuation system; **a** 3D model and **b** developed prototype

Most of the actuation systems present till date are either bulky or complex or compromise the performance of CTR [15]. To design a compact system, cascaded actuation modules are required. Due to the nesting of concentric tubes, miniaturized actuation system with cascaded design, and without pulleys and gears is difficult to manufacture using normal DC motors. In addition, the use of customized pulleys and gear systems may compromise the accuracy and incur backlash to the system. In this work, we propose a novel actuation system (shown in Fig. 4) using Hollow Shaft Nema8 stepper motors for the first time for CTR. Hollow shaft motors provide a big advantage of developing cascaded modules without integration of any other complex system. The tubes can be grasped near the proximal end and the rest of it can be passed through the hollow shaft. In this way, the members of CTR will stay in line minimizing chances of buckling and torsional windup. The members can be translated and rotated freely without compromising the degrees of freedom.

In this work, two modules were developed for driving two segments of the puncturing needle with each having 2-DOF. A total of four motors were used: two for translation and two for rotation. A lead screw assembly was attached to two motor’s shafts to convert the rotational motion into translational one. M4 × 0.7 and M6 × 1 screws with corresponding nuts were used for translation in modules of cannula and stylet, respectively. An aluminum linear rail with two sliders was attached to the base of the actuation system. The translating bodies (shown in Fig. 4) are an assembly of hollow shaft motor, attached to the nut and screwed to the slider of linear rail for each module. These translating bodies will move forward and backward by rotation of lead screws. The members of the puncturing needle will be attached to the shaft of the hollow shaft motor on the translating body. These motors will be responsible for providing a rotational degree of freedom. The base plate and motor attachment parts were manufactured from acetal to keep the assembly lightweight. Characteristics of the developed actuation system are presented in Table 2.

**Table 2:** Characteristics of the developed actuation system

Characteristics	Value
No. of actuators	4
Degrees of freedom	4
Modules	2
Size (*L* × *W* × *H*) (mm)	148 × 25 × 65
Available stroke (mm)	~ 100

#### Control

**Fig. 5:**
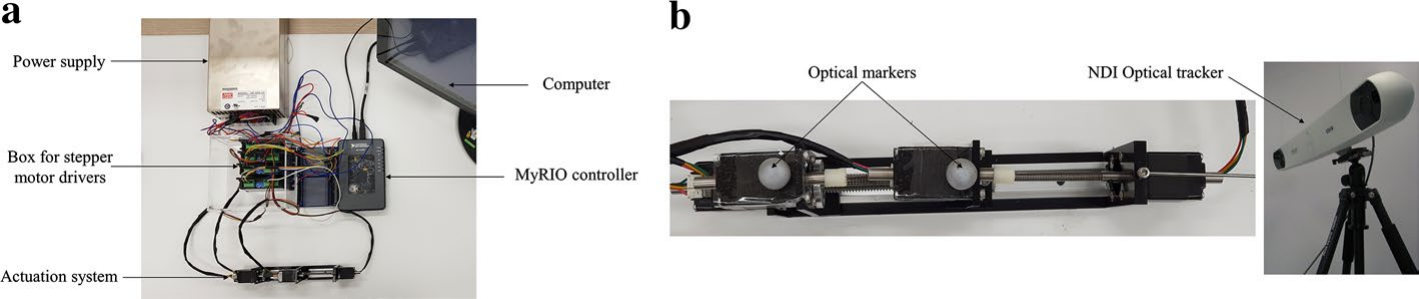
Experimental setup; **a** control setup for experimentations, **b** experimental setup to validate accuracy and repeatability of the actuation system

Four stepper motor drivers ‘Precise Step Drive’ (JT322L) provided by ROBOTDIGG were used to drive the motors. MyRIO controller from National Instruments Co. was used to control the rotation of the motors through a computer. At this stage, a simple position control PID controller was implemented in LabView program provided by National Instruments Co. The control setup used for experimentations is shown in Fig. 5a. A more precise controller for more accurate motion is left for future works.

## Results

Different sets of experiments were performed to validate the efficacy of the proposed needle system.

### Strain analysis

The shape optimization algorithm discussed before was used for solving the geometry of needle and provides parameters for shape setting. To validate the mechanical stability, strain induced in each segment because of the provided curvature value to needle segments is calculated. Equation 5 was used for calculating the maximum strain in individual segments:

5 $${\in} = - ky.$$

Here, *ϵ* is the maximum strain, *k* is the curvature, and *y* is the distance from neutral axis for each segment. By substituting values in Eq. 5, maximum strain in cannula and stylet was 0.0129 and 0.0050, respectively. As the nitinol material has an allowable strain limit of 11% [18], the optimized parameters of needle structure are mechanically stable.

### Accuracy and repeatability of the actuation system

The actuation system was designed to provide a stroke of 50 mm at both the cannula and stylet module. To validate it in the developed prototype, the accuracy of stroke was measured. An optical marker was attached on the top of the translating bodies of cannula and stylet module, as shown in Fig. 5b. The optical tracking system (OTS) from Northern Digital Inc. (NDI) Canada was used to measure the position of the optical marker attached to the moving body. The stroke for actuation and retraction was checked three times for both modules. The mean available stroke for cannula module was 49.78 mm and that for stylet module was 49.31 mm.

**Fig. 6:**
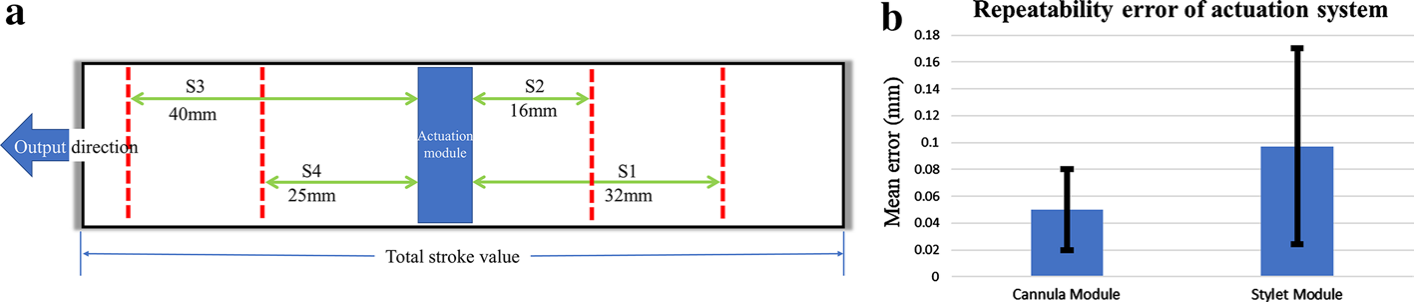
Repeatability experiments; **a** illustration of the movement used for repeatability experiments and **b** repeatability error plot in each module of the actuation system

The repeatability for translational motion of both modules was also checked using the same experimental setup. The translating body was initially moved to a random position (pivot position). Then, it was moved in one direction and brought back to the same pivot position. The translating body was moved four times to-and-fro from the pivot position; twice forward and twice backward. Each motion is repeated three times and the experimental technique is schematically shown in Fig. 6a. Using the information from OTS, error between the position of initial pivot position and position of pivot point after completing the stroke was determined. The average of mean error in case of cannula module was 0.050 mm, while that in case of stylet was 0.097 mm. The detailed results with standard deviations in each case are presented in Table 3 where all values are in mm and the error bar plot is shown in Fig. 6b. The second entry of Table 3 (S2) had the minimum stroke value, which can be responsible for the small error value when compared to others. In addition, the values were recorded using the OTS system and the values of S2 may have been measured when the fluctuation was the least. Mainly, all the error values are in submillimeter validating the claim that the presented actuation system can produce repeatable motions at the submillimeter scale for the translational case. Accuracy and repeatability analysis for rotational motion of modules is left for future works.

**Table 3:** Results of repeatability experiments (all values are in mm)

Cannula module					Stylet module			
Stroke no.	Test 1	Test 2	Test 3	Mean (*x̄*)	Test 4	Test 5	Test 6	Mean (*x̄*)
S1	0.001	0.044	0.240	0.095	0.188	0.261	0.122	0.190
S2	0.002	0.080	0.002	0.028	0.021	0.031	0.005	0.019
S3	0.002	0.048	0.088	0.046	0.020	0.045	0.135	0.067
S4	0.003	0.057	0.041	0.033	0.018	0.301	0.021	0.113
	Mean repeatability error			0.050	Mean repeatability error			0.097
	Standard deviation			0.030	Standard deviation			0.073

### Targeting accuracy and workspace of needle

**Fig. 7:**
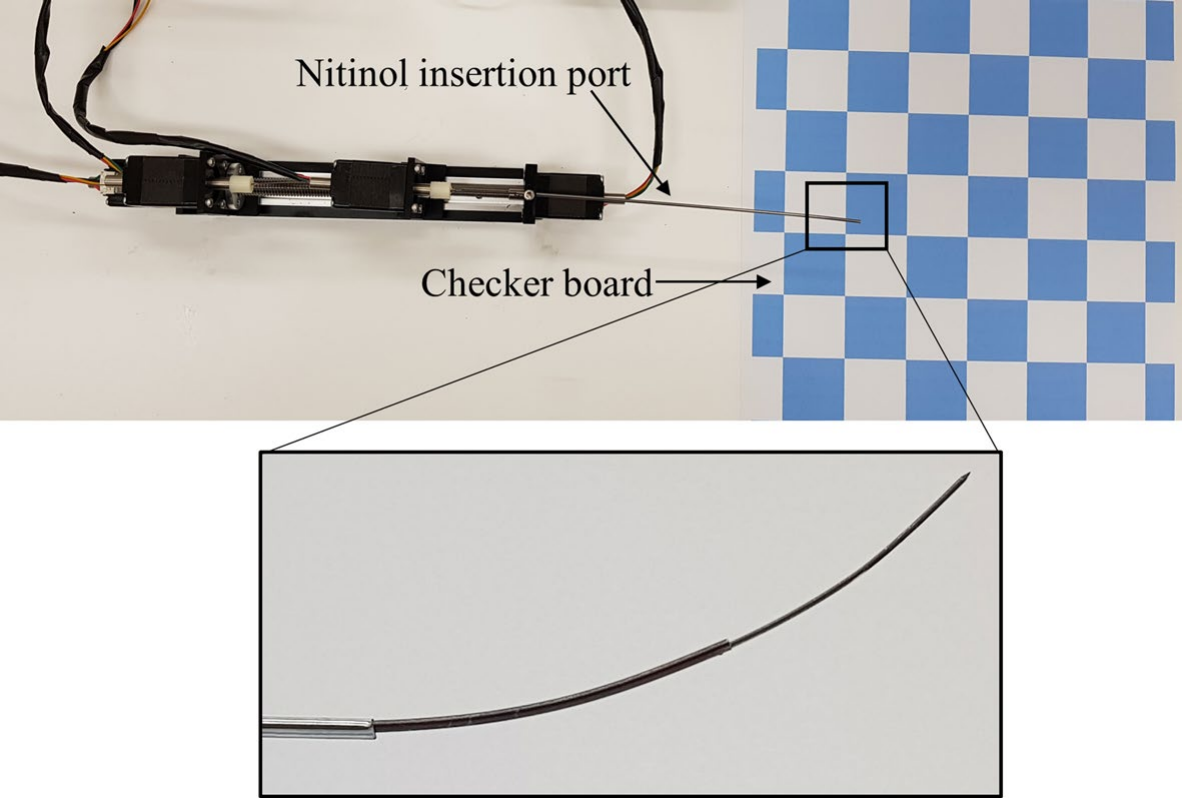
Experimental setup for targeting accuracy experiments for the needle. Inset view shows the final moved shape

**Fig. 8:**
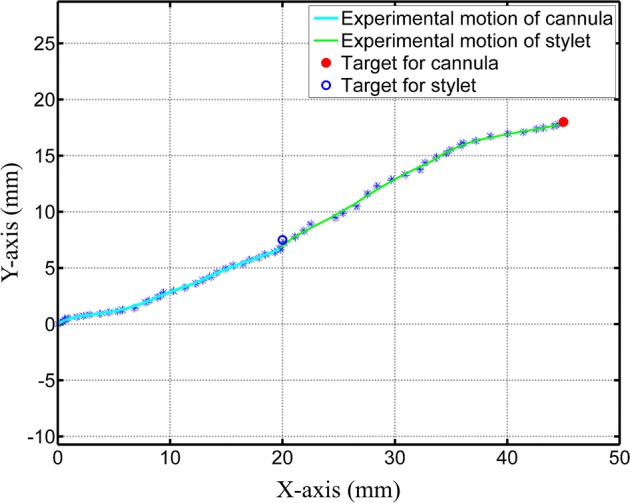
Real-time movement of the needle to reach target points

The developed prototype of the needle was analyzed to reach its optimized target in real time and error was computed. The experimental setup for checking the targeting accuracy of the needle is shown in Fig. 7. A checkerboard with 20 mm × 20 mm square boxes was placed underside of the needle and camera was mounted on the top. The pre-curved lengths of cannula and stylet were actuated from the insertion port and video was recorded using the camera. This video was analyzed in MATLAB^®^ to find the tip trajectory for real-time motion. The recorded video had a total of 2126 frames and pixel points of the tip of the needle were recorded after every 20 frames. Using the pixel difference from the checkerboard edges, millimeters per pixel value was computed which lead to the real-time tip position in 2D coordinates. These 2D coordinates are smoothed and real-time motion of the needle is shown in Fig. 8. The pixel points of moving tip were determined manually from the still frames of the video. This can be the reason for the glitches in the real-time motion of needle shown in Fig. 8. The targets for cannula and stylet tip provided in optimization algorithm were (20, 7.5) and (45, 18), respectively. The mean real-time targets achieved for cannula and stylet were (19.71, 6.70) and (44.58, 17.78), respectively. The overall error in targeting was computed by subtracting the positions of experimental and optimized target points. In the case of cannula and stylet, the mean targeting error was 0.55 mm and 0.48 mm, respectively. These results validate that the accuracy of submillimeter level can be achieved for target positioning of the needle using the current control algorithm and actuation system.

**Fig. 9:**
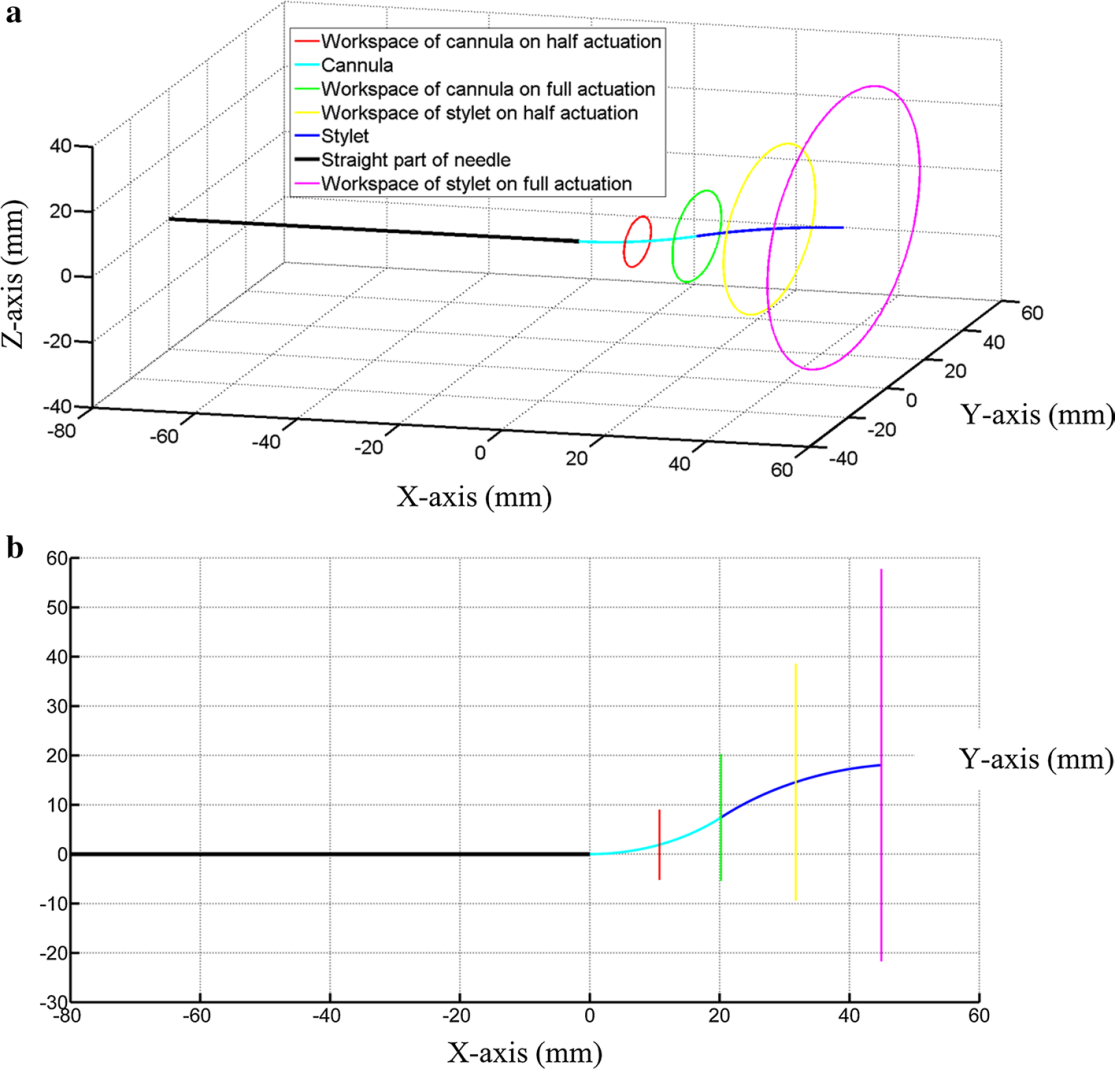
Workspace covered by the needle; **a** isometric view and **b** top view

In addition to targeting accuracy, the actual workspace covered by the needle in free space was also measured. The same experimental setup was used for workspace analysis, but, in this case, two cameras (one for the top view and one for side view) were mounted for video recording. Half of the pre-curved length of cannula segment was actuated from the insertion port and rotated around its axis. Using image processing, the position of the rotating cannula tip was tracked. Afterward, the whole pre-curved length of cannula body was actuated and rotated. Similarly, the stylet was initially actuated half and rotated like in case of cannula, but, here, the cannula was also rotated to get the combined workspace at the tip. Subsequently, the full length of stylet was actuated, and both segments were rotated and analyzed for subtended workspace. The obtained data points were fit to a circle and smoothen. The subtended workspace by the whole needle is shown in Fig. 9.

**Fig. 10:**
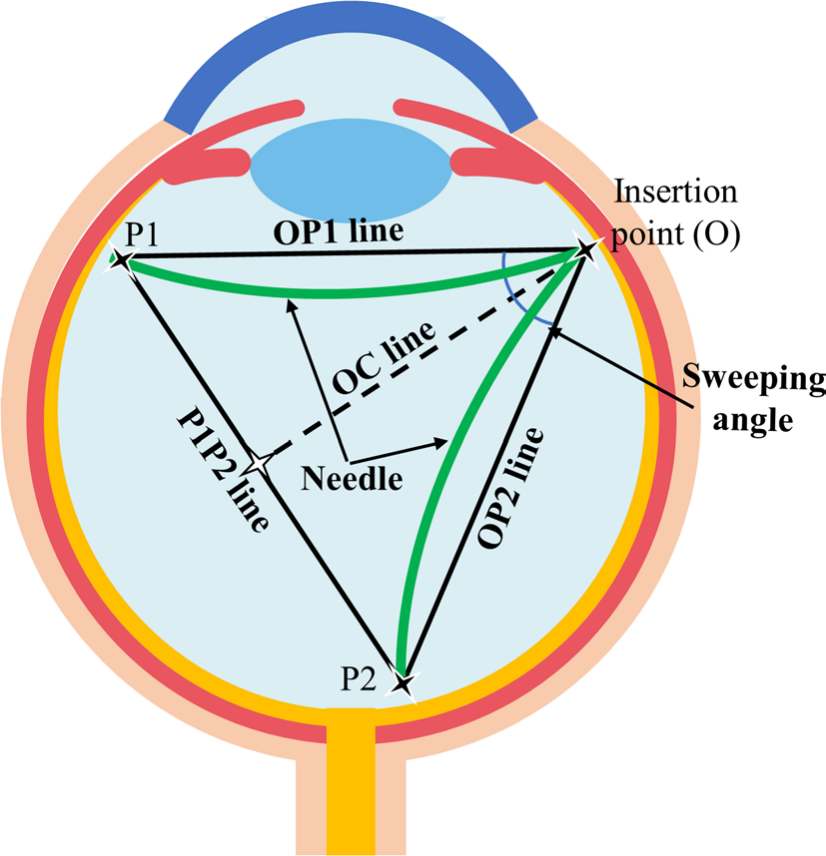
Geometrical method used for the calculation of the sweeping angle

Using the data of subtended workspace, the maximum angle swept by the needle was also calculated. A triangle was created using two lines (OP1 and OP2) from the farthest moved points (P1 and P2) by the needle tip to the insertion point (O) and by joining these farthest moved points by the line P1P2, as shown in Fig. 10. The shortest line OC from point O on the line P1P2 was determined (as it will be perpendicular on line P1P2 from point O). Trigonometric values were used, and the angle swept by the needle was computed. The presented needle system can sweep an angle of 128.5° inside the eyeball which is more than the concentric tube robot presented in [7] for use in vitreoretinal surgery. Hence, the proposed needle system improves the overall design of concentric tube robots for ophthalmic surgeries.

### Puncturing force

**Fig. 11:**
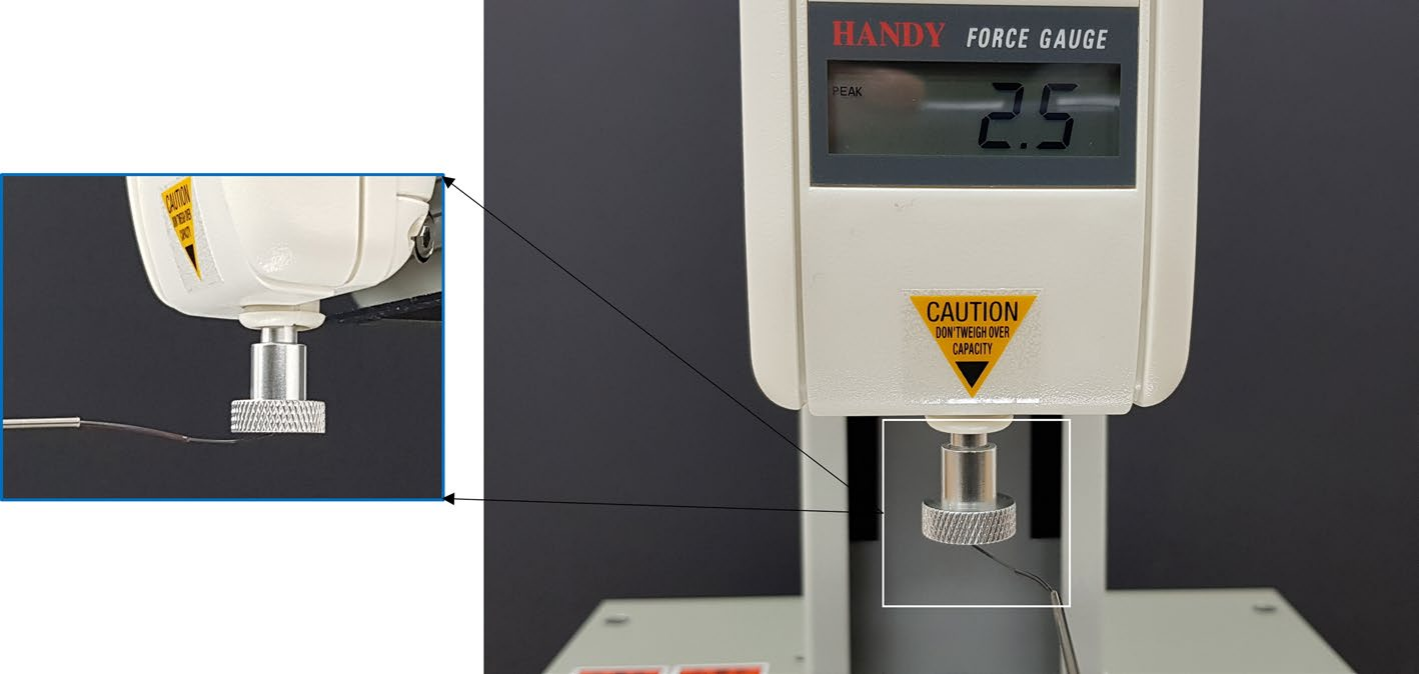
Maximum force generated by the puncturing tip only by the movement of the stylet. Inset view shows the contact point of the stylet tip with the load cell sensor (the load cell provides values in gram-force)

A puncturing force of around 15 mN is required for tasks of vein puncturing and membrane peeling in retinal surgeries [10, 19]. To use the presented needle for puncturing tasks, the force exerted by the laser-cut stylet tip was measured using a universal testing machine (JSV-1000) from Japanese Instrumentation System Co. (JISC, Japan). The attached load cell HF-1 from JISC LTD had accuracy in millinewtons. The puncturing tip was brought in contact with the sensor of load cell by actuation and retraction of stylet using motors. The maximum payload which it can provide for puncturing was around 24.50 mN (shown in Fig. 11). The test was repeated three times and mean force produced for puncturing was 23.51 mN with a standard deviation of ± 0.81 mN. Thus, the laser-cut stylet tip provides the necessary force to carry out surgical tasks inside the eye.

## Discussion

To avoid stitching of sclera in vitreoretinal surgeries, the procedure should be carried out by a minimum number of incisions and smaller tool diameters. The currently available straight tools for vitrectomy have two major limitations. (1) If there are multiple surgical targets, the straight tools are required to be reinserted increasing the number of incisions. (2) In the case of distant targets, the surgeons push out the eye of the patient which cause pain and trauma. Therefore, the proposed needle system is bendable and does not need reinsertions if a new surgical target has to be reached. In addition, the relative motion of cannula and stylet can generate 4-DOF motion and can help in reaching distant targets. The needle system was also used in the albumen and different targets were accessed without any difficulty making it viable for vitreoretinal procedures. Along with the clinical advantages, the proposed needle system improves the CTR design used previously in [7] by increasing the swept workspace from 110° to 128.5°. This increase in the workspace is advantageous as the surgical target requires access to the distant parts of the eyeball. With the proposed design, the infectious arteries and veins can be accessed more easily without repeated insertions and the least amount of stress on the sclera. Regarding the actuation system for CTR, this work proposes a novel approach using hollow shaft motors for the first time. CTR require cascaded drive units and the hollow shaft motors are specifically advantageous for such cases. The concentrically arranged segments of CTR can be grasped and allowed to pass through at the same time. It was not possible before using only DC or servo motors and additional assemblies of gears were required which compromised the size and accuracy of the actuation system. In addition to that, the presented actuation system is more miniaturized, which provides better stroke value and accurate motions when compared with the already available solution of [15].

The presented prototype of needle and actuation system in this work is earlier versions and needs further developments. First, for operation in the eye, force sensing and tremor cancelation are very important. If the force exerted on the sclera and retinal structures is more than the permissible value, serious damage may occur. The stylet tip can generate more than the required 15 mN force which may be helpful to puncture the vessel, but it should be controlled to be in the range of the required value. To curtail this issue, force sensing is vital in any ophthalmic surgery robot, and the immediate future goal is to implement force sensing in both cannula and stylet. By limiting the applied force, the stylet will only puncture the vessel except for going through it and this will also avoid damage to the neighboring structures.

Second, the current version of the actuation system is already miniaturized, but surgeons mostly prefer handheld or joystick-operated devices. Therefore, a surgically ergonomic actuation system will be developed and a motor-based telerobotic actuation is the most desirable candidate till date.

Third, the designed prototype can only be used for puncturing tasks at this stage. By modifications in the actuation device, the stylet tip can also be used for membrane peeling. Another application can be on-site drug delivery after puncturing. For this purpose, the length of cannula should be increased, so that it can also reach the surgical target and the stylet should be retracted. The design of the proposed version of the needle system has a possibility to retract the stylet, but it is limited due to the current version of the actuation system. The future prototypes of the needle and actuation system will compensate for stylet retraction, so that the drug can be delivered through the cannula to the punctured vein.

Fourth, the optimization algorithm at this stage does not consider the torsional stability of segments and a more advanced optimization algorithm is in development. The inverse kinematics of the concentric tube structure will be solved too for more detailed analysis.

Fifth and last, if the incision in sclera is around 0.9 mm, the patient may require stitching after the surgical process. If the incision size is reduced to 0.6 mm or less, post-surgery stitching is not required. The current dimensions of the needle system lie in the gray area for stitching and will be miniaturized to be less than 0.6 mm in the future version.

In the end, experiments validated the potentials of the developed prototypes of puncturing needle for vitrectomy and the novel actuation system for concentric tube robots. The presented prototype of the needle can cover sufficient workspace inside the eyeball and exert tip force necessary for puncturing. The proposed actuation system is accurate and can produce repeatable motions. Further efficacy of the needle and actuation system will be validated by analysis of the future versions with above-mentioned changes.

## Conclusion

This work presents a new 4-DOF puncturing needle to maneuver behind the retina and reach distal targets with increased workspace to solve issues encountered by a straight tool. A concentric tube-based approach was used and the proposed 25G needle has 4-DOF with two pre-curved segments; cannula and stylet. To drive this concentric tube robot (CTR), a novel and miniaturized actuation using hollow shaft stepper motors was also developed. It provides a stroke of 100 mm in a small size of 148 × 25 × 65 mm (*L* × *W* × *H*).

The experimental results validated that the developed actuation system produces repeatable motions and the needle can reach the surgical targets with accuracy in submillimeter. It can cover an extended workspace of 128.5° which improves the design of CTR for eye surgery. The tip of stylet was specifically designed for puncturing tasks and provides a force of 23.51 ± 0.81 mN.

The future versions of presented systems will include a force sensor to limit the amount of force inside the eyeball. In addition, a surgically ergonomic actuation system will be developed to assist surgeons in clinical trials.
